# Supplementary figures and images for: Architecture of the biofilm-associated archaic Chaperone-Usher pilus CupE from Pseudomonas aeruginosa
Source: PLoS Pathog. 2023 Apr 14;19(4):e1011177. doi: 10.1371/journal.ppat.1011177 (PMC10104325; doi:10.1371/journal.ppat.1011177)

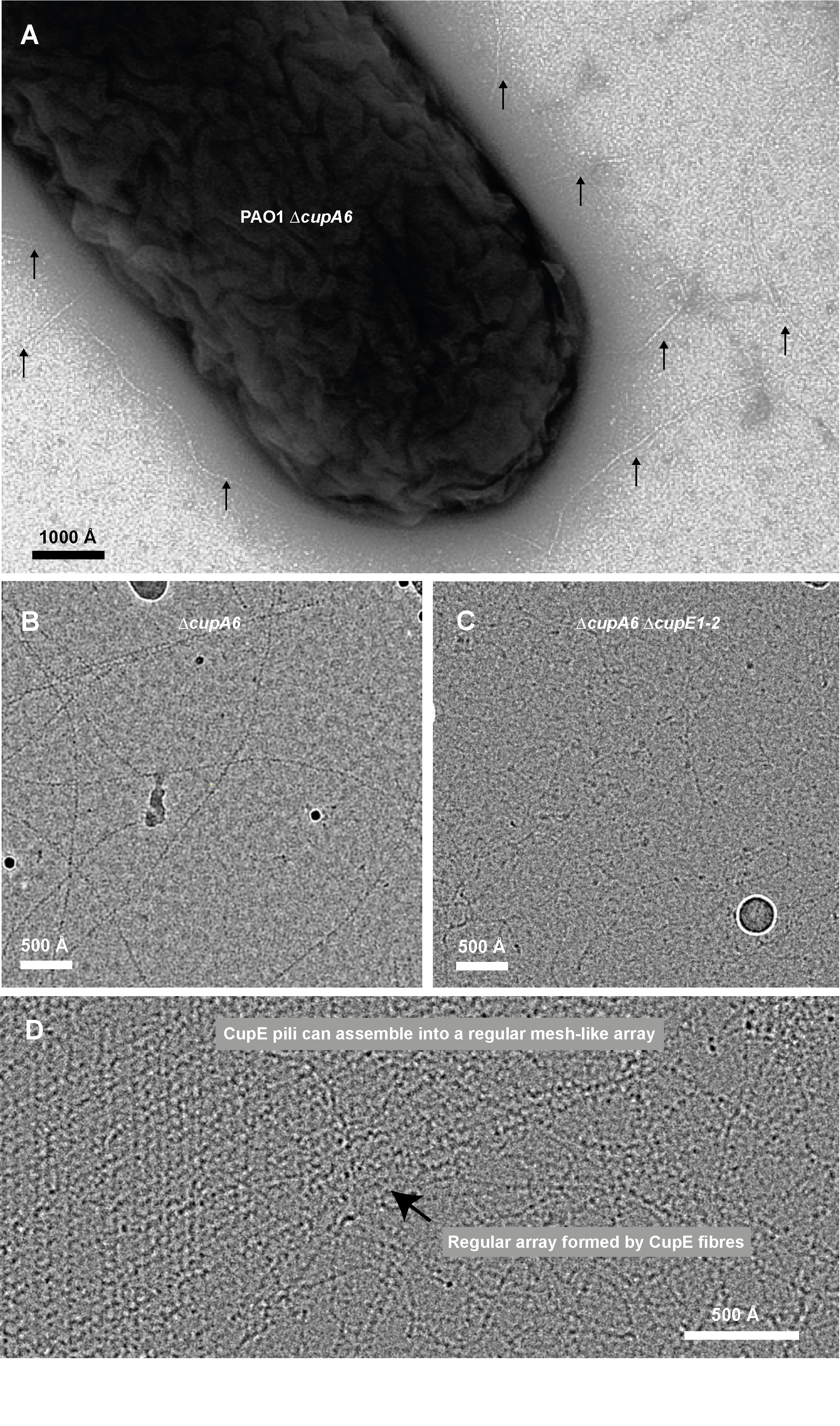

Supplement: S1 Fig — (A) Negative stain image of a P. aeruginosa PAO1 strain with cupA6 deletion. Deletion of cupA6 causes upregulation of cell-surface fibres compared to a control strain without the deletion (188 fibres in the ΔcupA6 strain versus 60 fibres in a control strain; n = 30 micrographs; see Methods). Fibres are highlighted by arrows. (B-C) Cell surface filaments were sheared, precipitated, and subjected to cryo-EM, showing (B) the ΔcupA6 strain as shown in (A), and (C) a ΔcupA6 ΔcupE1-2 strain, demonstrating that the cell surface pili with a dotted zigzag pattern are CupE pili. Small fibre contamination is enriched in (C). This contaminant is possibly DNA due to its size and low persistence length, which precipitates under similar conditions [77]. (D) In the cryo-EM dataset of purified CupE pili, instances of CupE pili forming a crisscross mesh-like array were observed. (TIF) [file ppat.1011177.s001.tif]

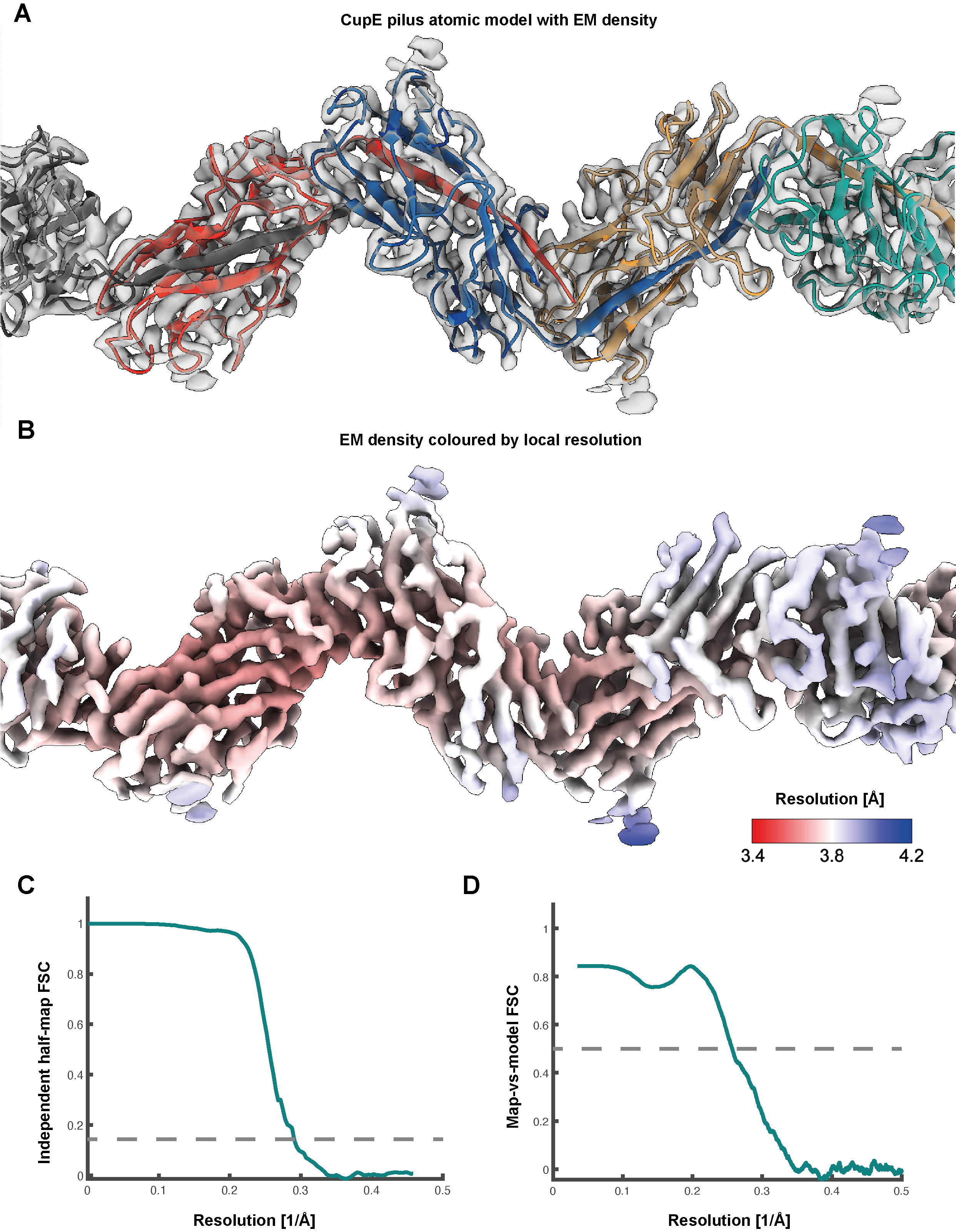

Supplement: S2 Fig — (A) Atomic model of the CupE pilus, consisting of CupE1 subunits, in the transparent cryo-EM density at 15 σ away from the mean. (B) The same density shown in (A) coloured according to local resolution. (C) Independent half-map FSC curve. (D) Map-vs-model FSC curve. (TIF) [file ppat.1011177.s002.tif]

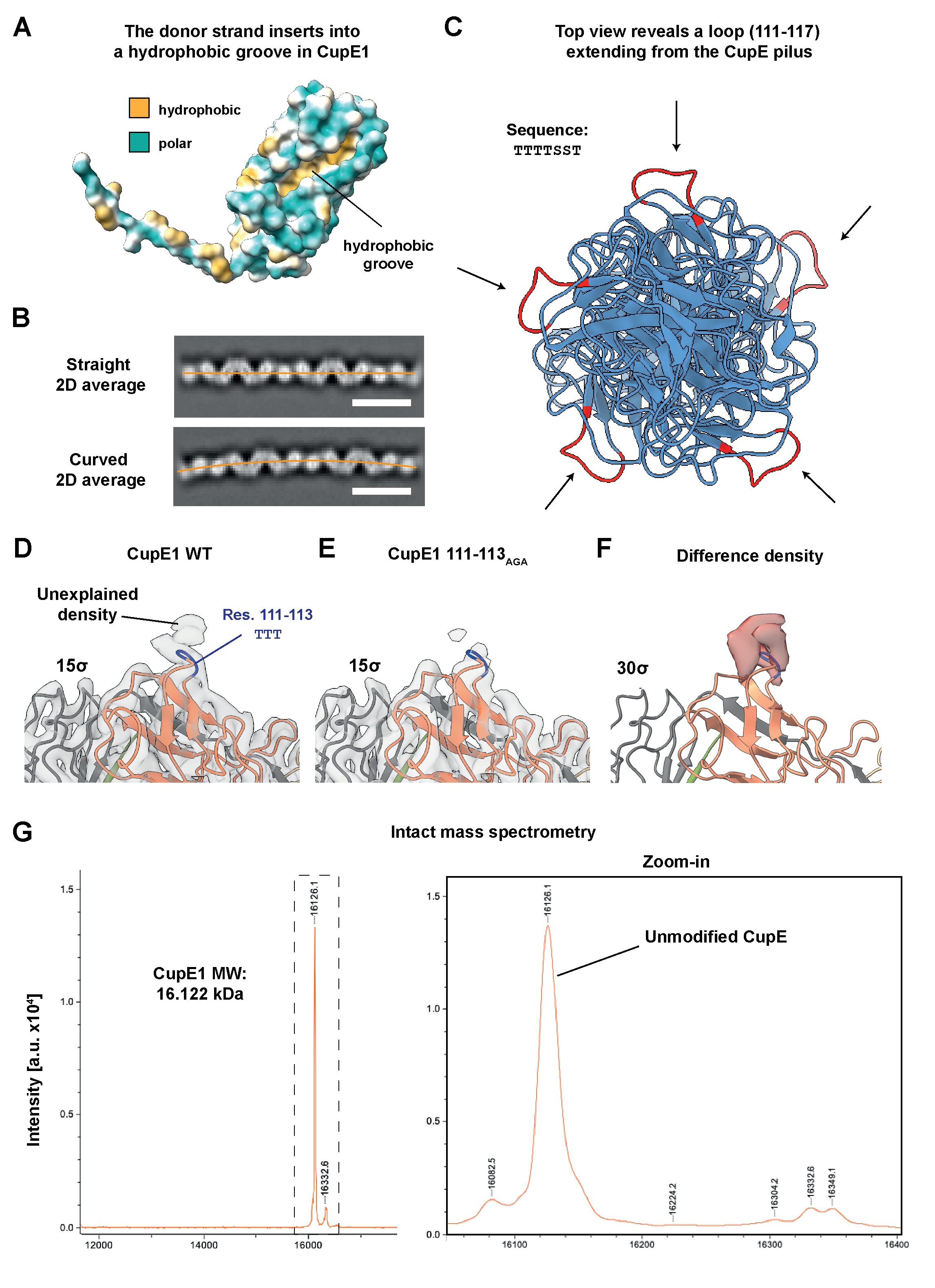

Supplement: S3 Fig — (A) Hydrophobic surface depiction of an uncomplemented CupE1 subunit reveals that the donor strand inserts into a hydrophobic groove. (B) Straight and curved 2D class averages of CupE pili. Orange lines indicate the center of the filament to facilitate the visualization of curvature. Scale bars are 100 Å. (C) Top view of a five-subunit ribbon model of the CupE pilus reveals that a serine-threonine-rich loop (marked red, sequence TTTTSST) extends from the pilus, exposed to the environment. (D-F) Mutation of the first three residues of the loop shown in (C) to AGA (111-113AGA) followed by structural determination at 4.1 Å resolution via cryo-EM shows reduced density near the loop, suggesting this density could arise from post-translational modifications. Density is shown at 15 σ contour level in (D) and (E), difference density is shown at 30 σ in (F). (G) Intact MALDI mass spectrometry of CupE preparations. Samples were treated 1:1 with 70% formic acid to trigger disassembly into monomers and spotted 1:1 with sinapinic acid. The major peak (16.126 kDa) corresponds to the predicted weight of CupE1 (16.122 kDa). (TIF) [file ppat.1011177.s003.tif]

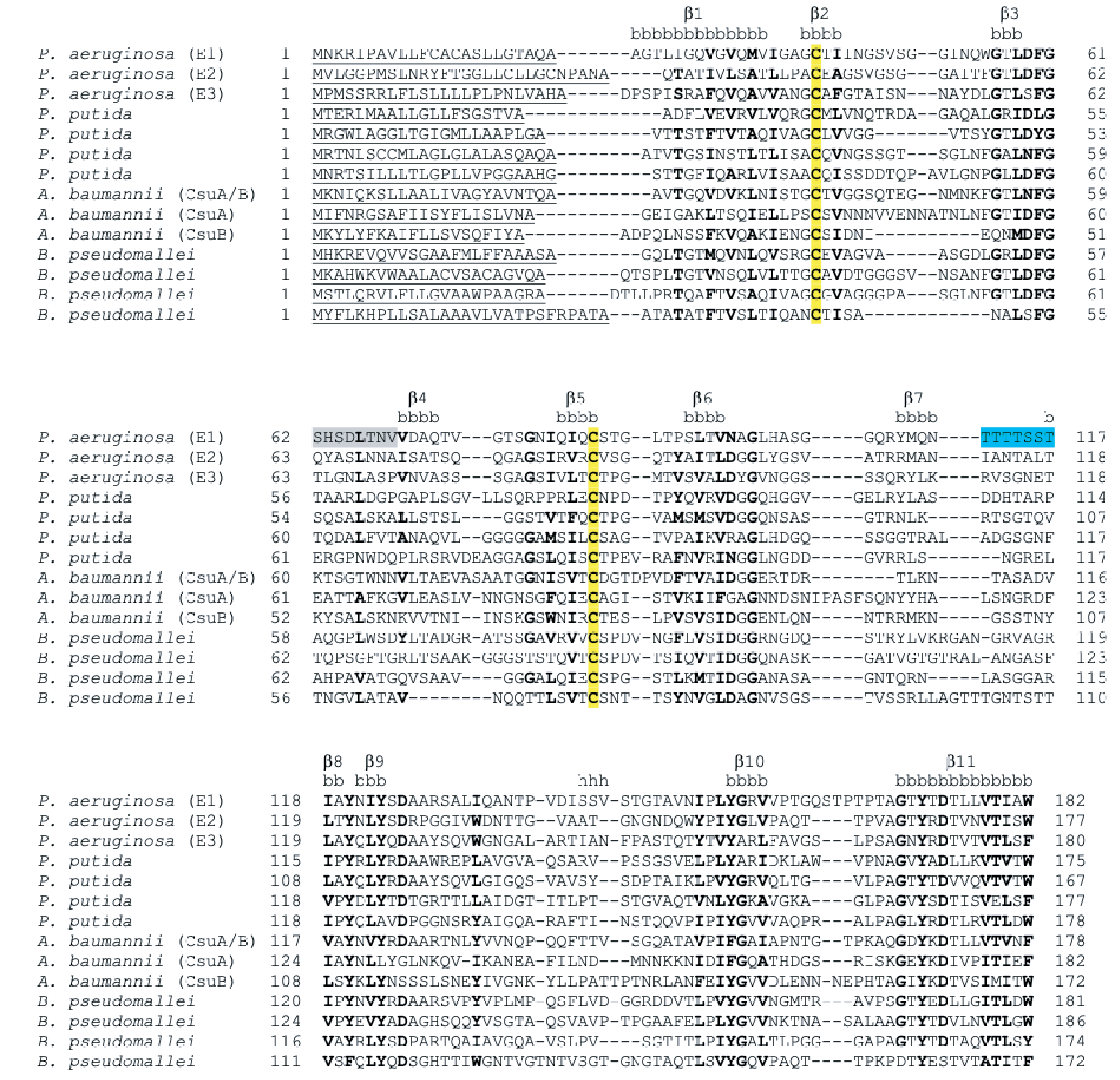

Supplement: S4 Fig — Conserved residues are shown in boldface. Secondary structure (b = β-strand, h = α-helix) is annotated based on our cryo-EM structure of the CupE1 filament. The conserved cysteine residues (C41 and C85) that form a disulfide bond in CupE1 filaments are highlighted in yellow and the serine-threonine-rich loop in cyan. The signal peptide in each sequence, as predicted using SignalP 6.0 [75], is underlined. Accession details for the shown protein sequences are provided in the S5 Table. Residues 62–69, which are located near the subunit-subunit interface, are marked in grey. L66 and V70, which interact with the donor strand within the same subunit fold, are conserved; residues facing the subunit-subunit interface are not conserved. (TIF) [file ppat.1011177.s004.tif]

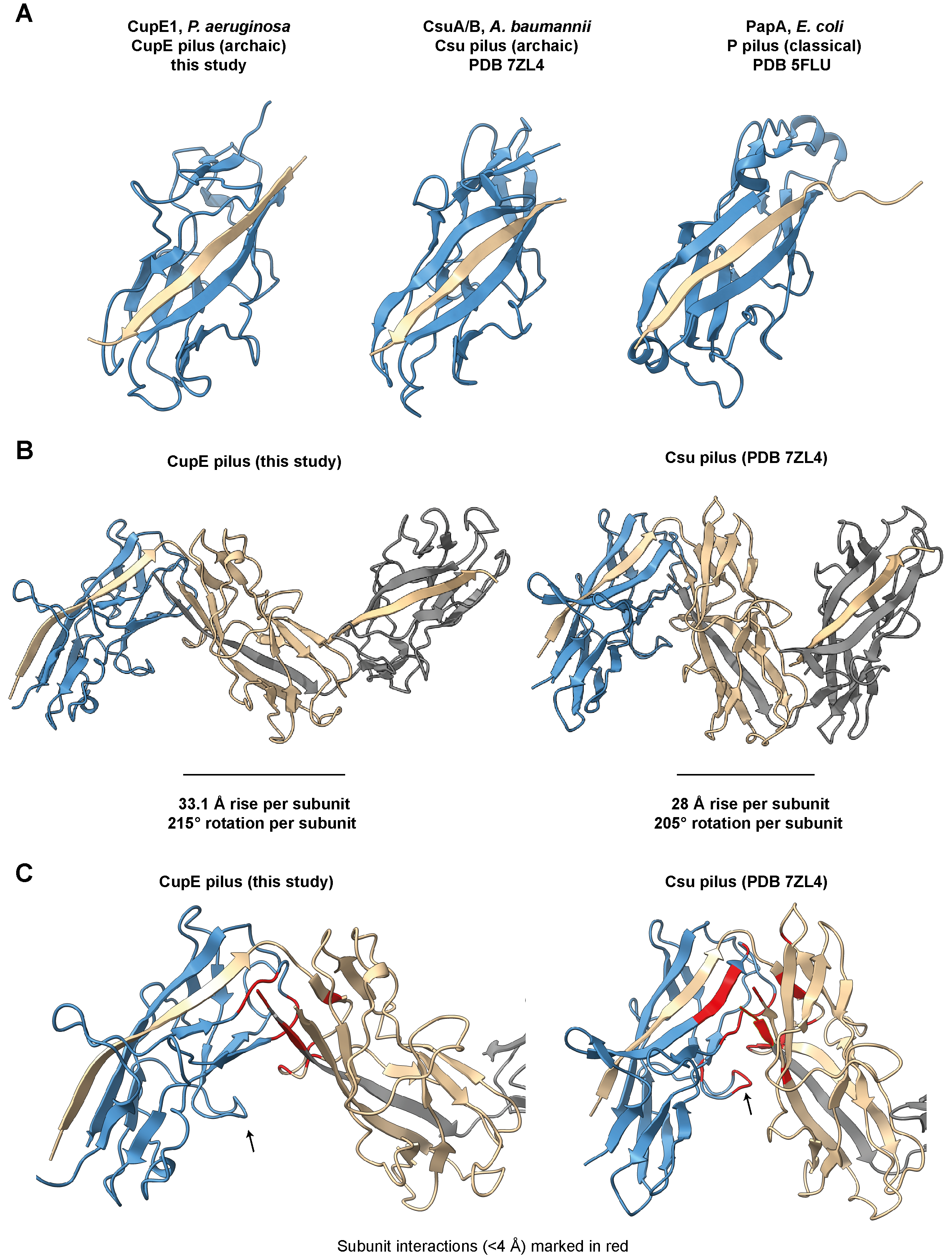

Supplement: S5 Fig — (A) Comparison of the CupE1 subunit with CsuA/B (PDB 7ZL4, RMSD 1.545 Å) and PapA (PDB 5FLU, RMSD 5.21 Å). (B) Pilus architecture of the CupE pilus versus the Csu pilus. (C) Subunit contacts (<4 Å interaction) between main pilins within the CupE and Csu pilus. Interacting residues are marked in red. A loop contributing to the subunit interface in the Csu pilus, but not in the CupE pilus, is marked with an arrow. (TIF) [file ppat.1011177.s005.tif]

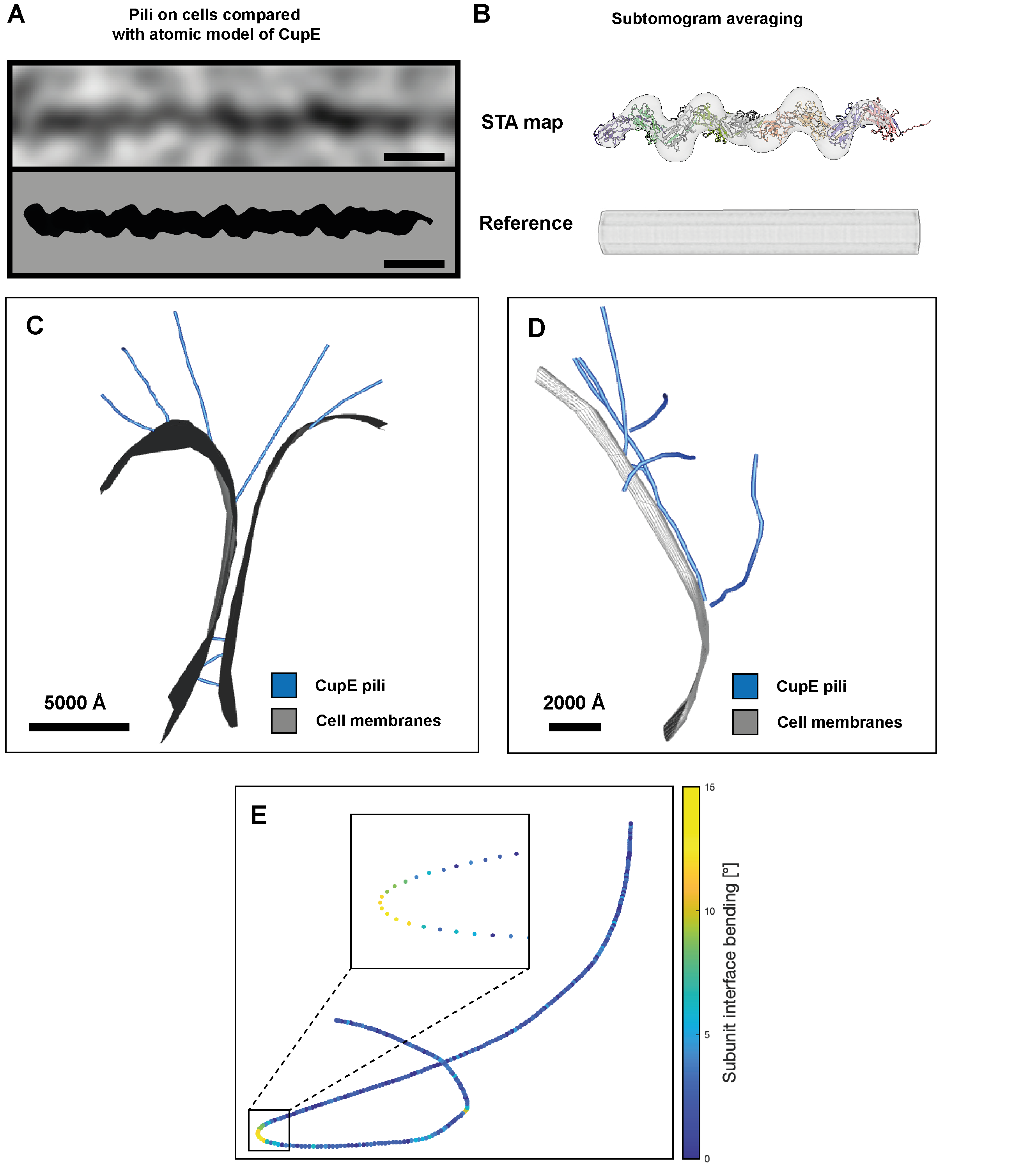

Supplement: S6 Fig — (A) Cryo-ET of pili on cells (upper) recaptures the size and zigzag architecture of the atomic model of CupE, which was projected at 10 Å resolution for comparison (lower). Scale bar is 100 Å. (B) Subtomogram averaging of pili on cells results in zigzag-shaped density consistent with the atomic model shown as ribbons. Particles were aligned against a cylindrical reference to prevent bias. (C-D) Segmentation of tomograms as shown in Fig 3. (E) Quantification of local pilus curvature, by measuring angular deviation from the helical axis of the pilus on a P. aeruginosa cell (shown in Fig 3C). (TIF) [file ppat.1011177.s006.tif]

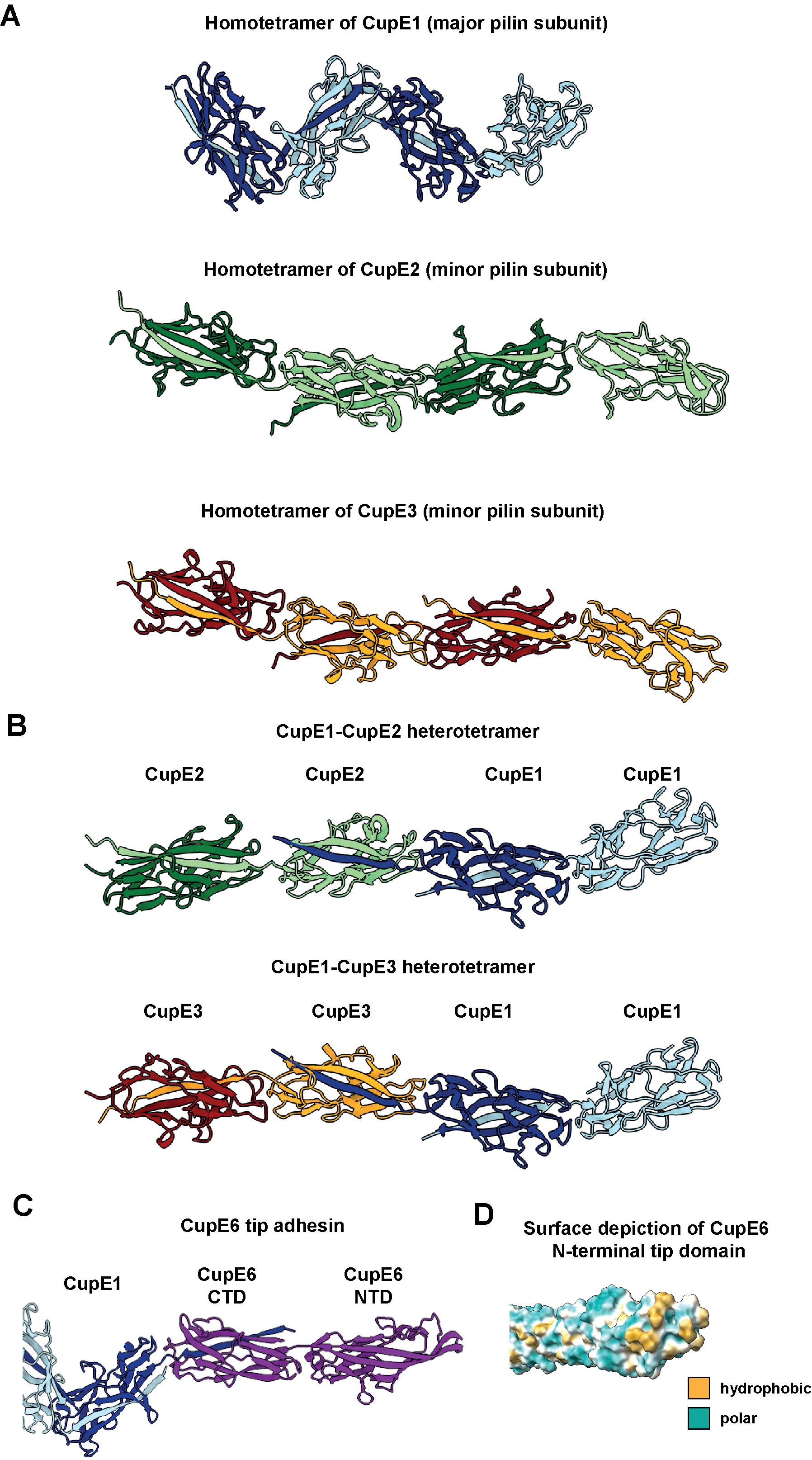

Supplement: S7 Fig — (A) Predictions of homotetramers of CupE1, CupE2, and CupE3. The model for CupE1 was validated by comparison with the cryo-EM structure (Cα-RMSD of E1/E1 subunits: 0.96 Å) (Figs 1 and 2). All pilins of CupE are predicted to share a similar structure (Cα-RMSD E1/E2: 1.64 Å, E1/E3: 1.63 Å, E2/E3: 0.89 Å), and CupE2 and CupE3 are also predicted to polymerize through donor-strand complementation. Pilins mainly differ in domain orientation within the filament. (B) Predictions of heterotetramers consisting of two CupE1 and CupE2 subunits (upper) or two CupE1 and two CupE3 subunits (lower). The modelling suggests that donor strand exchange is possible between major and minor pilin subunits but does not reveal a preferred subunit arrangement. (C) Prediction of a filament consisting of CupE1 capped with the CupE6 adhesin tip subunit. The adhesin protein CupE6 consists of two domains, with the C-terminal domain capping the filament and the N-terminal domain exhibiting hydrophobic surface patches as predicted in previous studies [19]. (D) CupE6 adhesin domain prediction as in (B) shown as hydrophobic surface depiction, revealing a hydrophobic patch at the domain tip. (TIF) [file ppat.1011177.s007.tif]

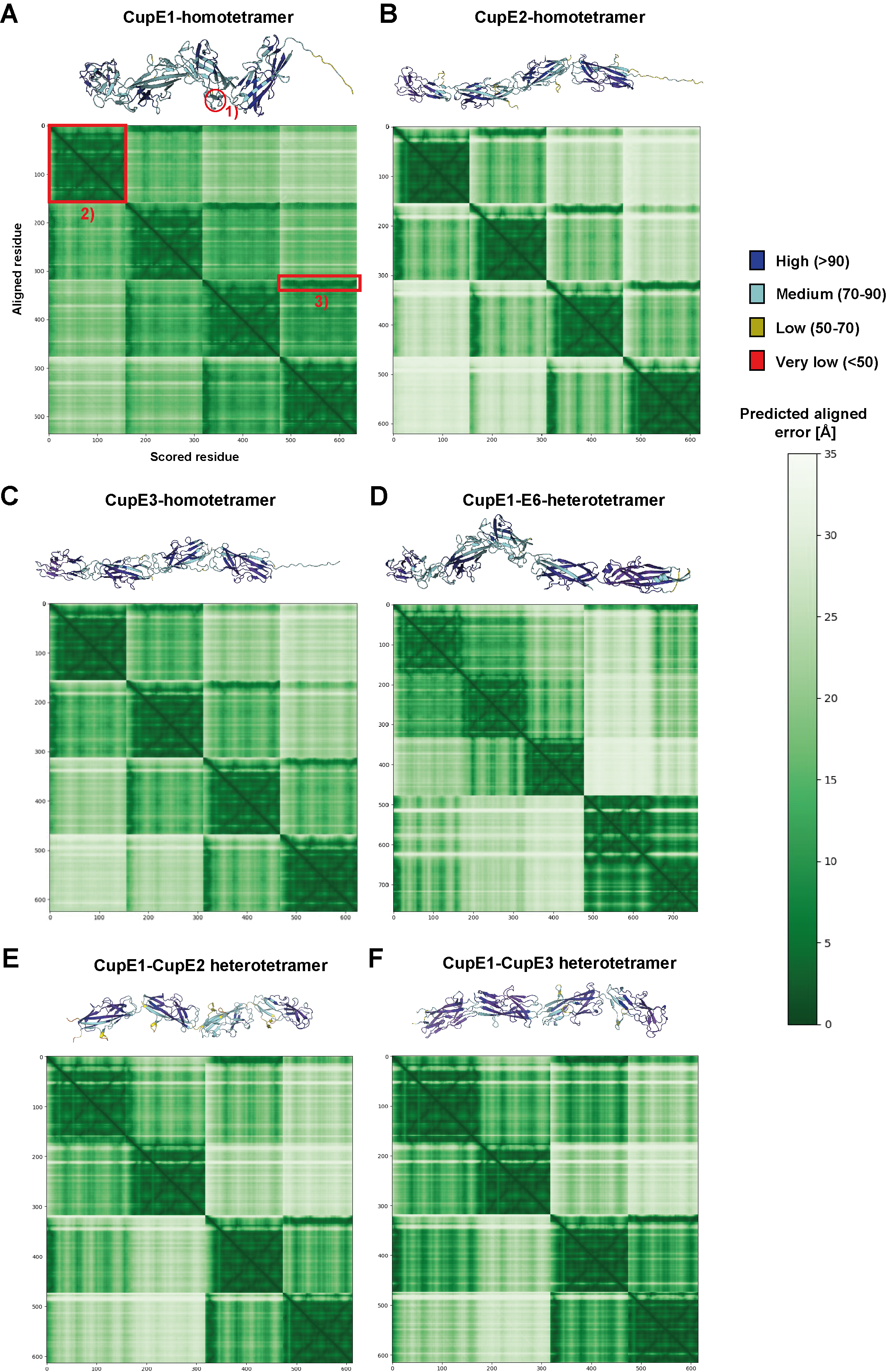

Supplement: S8 Fig — The prediction confidence is evaluated with different scores. Structures are colored according to their predicted Local Distance Difference Test (pLDDT). The pLDDT measures the model confidence per residue [44]. The pLDDT for all tetramers is very high or high for most parts of the prediction and low only for β-hairpin motifs (e.g. labeled as ‘1’ in (A)). The PAE (Predicted Aligned Error) measures the expected positional error (in Å) at residue X, when the predicted and true structures are aligned on residue Y [44,78]. The PAE is visualized as a heatmap, where green means low expected error, showing the PAE for every pair of residues, resulting in 4x4 submatrices for every PAE heatmap. Every submatrix (e.g., labeled as ‘2’) on the diagonal shows the intra-subunit PAE. It shows that the intra-domain PAE for all filaments and the PAE for the donor β-strand interaction (e.g., labeled as ‘3’) is low, arguing that the protein structure for one filament subunit is predicted correctly. The inter-subunit predicted error increases when the distance between the subunits is greater. This implies on the one hand that the inter-subunit orientation prediction should not be taken as a precise measurement. On the other hand, it could mean that the filament is flexible and therefore no rigid inter-subunit conformation exists, consistent with our cryo-EM data. In (D), the relative domain position within the CupE6 adhesin is predicted with high confidence, but the PAE for the relative orientation of CupE6 against the CupE1 filament is high. This could again mean high flexibility, but also that the predicted adhesin orientation must be interpreted with care. (TIF) [file ppat.1011177.s008.tif]
