# Supplementary material for: Architecture of the biofilm-associated archaic Chaperone-Usher pilus CupE from Pseudomonas aeruginosa
Source: PLoS Pathog. 2023 Apr 14;19(4):e1011177. doi: 10.1371/journal.ppat.1011177 (PMC10104325; doi:10.1371/journal.ppat.1011177)
Supplement: S1 Table — (DOCX) [file ppat.1011177.s009.docx]

| **Data collection and processing** | CupE1 WT [EMDB-16683, PDB 8CIO] | CupE1 111-113_AGA_ [EMDB-16686] |
| --- | --- | --- |
| Microscope | Krios Titan G3 | Krios Titan G3 |
| Magnification | 81,000 | 81,000 |
| Voltage (kV) | 300 | 300 |
| Electron exposure (e^–^/Å^2^) | 46 | 45 |
| Defocus range (μm) | -1 to -2.5 | -1 to -2.5 |
| Pixel size (Å) | 0.546 (super-resolution)  1.092 (physical, final) | 0.546 (super-resolution)  1.092 (physical, final) |
| Symmetry imposed | Helical, final: 214.56° 33.12 Å | Helical, final: 214.67°, 33.04 Å |
| Initial particle images (no.) | 3,766,858 | 1,259,369 |
| Final particle images (no.) | 274,457 | 88,886 |
| Map resolution (Å)  FSC threshold | 3.47  0.143 | 4.13  0.143 |
| Map resolution range (Å) | 3.4-4.2 | 3.8-4.7 |
|  |  |  |
| **Refinement** |  | No atomic model built |
| Initial model used (PDB code) | n/a |  |
| Model resolution (Å)  FSC threshold | 2.9/3.1/3.9  0/0.143/0.5 |  |
| Model resolution range (Å) | n/a |  |
| Map sharpening *B* factor (Å^2^) | -97 | -152 |
| Model composition  Non-hydrogen atoms  Protein residues  Ligands | 5655  795  0 |  |
| *B* factors (Å^2^)  Protein  Ligand | 66.88  n/a |  |
| R.m.s. deviations  Bond lengths (Å)  Bond angles (°) | 0.002  0.547 |  |
| Validation  MolProbity score  Clashscore  Poor rotamers (%) | 1.95  8.03  0 |  |
| Ramachandran plot  Favored (%)  Allowed (%)  Disallowed (%) | 91.08  8.92  0 |  |
