# Supplementary material for: Architecture of the biofilm-associated archaic Chaperone-Usher pilus CupE from Pseudomonas aeruginosa
Source: PLoS Pathog. 2023 Apr 14;19(4):e1011177. doi: 10.1371/journal.ppat.1011177 (PMC10104325; doi:10.1371/journal.ppat.1011177)
Supplement: S2 Table — (DOCX) [file ppat.1011177.s010.docx]

| **Construct** | **Primer name** | **Sequence** |
| --- | --- | --- |
| pKNG-(∆*cupA6*) | ∆*cupA6* P1 | AACCGGATACCGCGCTGGC |
|  | ∆*cupA6* P2 | TCACCCCTGACCGTTCACTGTAGAGC |
|  | ∆*cupA6* P3 | GTGAACGGTCAGGGGTGACCGGGGAGG |
|  | ∆*cupA6* P4 | CCTGGGTGAACAGTTCCACG |
|  | ∆*cupA6* P5 | GAGAAACTCGCGCTCGGCAG |
|  | ∆*cupA6* P6 | AGTACCAGCAACCACAGGCTG |
| pKNG-(∆*cupE1-2*) | ∆*cupE1-2* P1 | CTGCTGTTCGGCACCATCGCCTC |
|  | ∆*cupE1-2* P2 | GGCGGTGTTCAACAAGACTGCCGGAAT |
|  | ∆*cupE1-2* P3 | GTCTTGTTGAACACCGCCCTCACCCTG |
|  | ∆*cupE1-2* P4 | CTGCTGCTGCCGCCATTGA |
|  | ∆*cupE1-2* P5 | GCCATCACCCTGGCCTTC |
|  | ∆*cupE1-2* P6 | TTGCCCTCCCAGCGGACAT |
| pKNG-(CupE1-2 AGATSST) | CupE1 AGATSST Fw | AGCGGCGGCCAGCGCTACATGCAGAACGCCGGCGCCGGCGCCGGCGCCATCGCCTACAACATCTATTCGGACG |
|  | CupE1 AGATSST Rev | CGTCCGAATAGATGTTGTAGGCGATGGCGCCGGCGCCGGCGCCGGCGTTCTGCATGTAGCGCTGGCCGCCGCT |
