# Supplementary material for: Architecture of the biofilm-associated archaic Chaperone-Usher pilus CupE from Pseudomonas aeruginosa
Source: PLoS Pathog. 2023 Apr 14;19(4):e1011177. doi: 10.1371/journal.ppat.1011177 (PMC10104325; doi:10.1371/journal.ppat.1011177)
Supplement: S3 Table — (DOCX) [file ppat.1011177.s011.docx]

| **Plasmid** | **Characteristics** | **Source** |
| --- | --- | --- |
| Cloning vectors | | |
| pCR-Blunt II-TOPO | Sub-cloning vector for constructs synthesized by KOD PCR, (Km^R^) | Invitrogen |
| pRK2013 | Self-transmissible helper plasmid for three- partner conjugations, Km^R^ | (1) |
| Chromosomal mutagenesis vectors | | |
| pKNG101 | Non-replicative suicide vector for *P. aeruginosa* chromosome mutagenesis, ori6K, mobRK2, *sacB* gene for sucrose sensitivity, Sm^R^ | (2) |
| pKNG-(∆*cupA6*) | Suicide vector to delete *cupA6* (PA2133) from *P. aeruginosa*, Sm^R^ | This study |
| pKNG-(∆*cupE1-2*) | Suicide vector to delete *cupE1-2* (PA4648-9) from *P. aeruginosa*, Sm^R^ | This study |
| pKNG-(CupE1-2 AGATSST) | Introduction of mutation of threonine-rich loop TTTTSST to AGATSST in CupE1 | This study |

**References**

1. Figurski DH, Helinski DR. Replication of an origin-containing derivative of plasmid RK2 dependent on a plasmid function provided in trans. Proceedings of the National Academy of Sciences. 1979;76(4):1648-52.

2. Kaniga K, Delor I, Cornelis GR. A wide-host-range suicide vector for improving reverse genetics in gram-negative bacteria: inactivation of the blaA gene of Yersinia enterocolitica. Gene. 1991;109(1):137-41.
