# Supplementary material for: Architecture of the biofilm-associated archaic Chaperone-Usher pilus CupE from Pseudomonas aeruginosa
Source: PLoS Pathog. 2023 Apr 14;19(4):e1011177. doi: 10.1371/journal.ppat.1011177 (PMC10104325; doi:10.1371/journal.ppat.1011177)
Supplement: S4 Table — (DOCX) [file ppat.1011177.s012.docx]

| **Strain** | **Relevant features** | **Reference** |
| --- | --- | --- |
| *E. coli* |  |  |
| DH5α | Strain used for cloning. F– endA1 glnV44 thi- 1 recA1 relA1 gyrA96 deoR nupG purB20 Φ80dlacZΔM15 Δ(lacZYA-argF)U169, *hsd*R17(r_K_^-^, mK^+^), λ^-^ | Invitrogen |
| CC118(λpir) | Strain used for pKNG101 maintenance. Δ(*ara*-*leu*) araD ΔlacX74 galE galK-phoA20 thi-1 rpsE rpoB argE (Ap^R^) recA1 Rfr λpir. | (1) |
| *P. aeruginosa* |  |  |
| PAO1 Δ*pilA* Δ*fliC* Δ*mvaT* | Deletion of *pilA* (PA4525); *fliC* (PA1092) and *mvaT* (PA4315) | Eleni Manoli, laboratory collection |
| PAO1 Δ*pilA* Δ*fliC* Δ*mvaT* Δ*cupA6* | Deletion of *pilA* (PA4525); *fliC* (PA1092); *mvaT* (PA4315) and *cupA6* (PA2133) | This study |
| PAO1 Δ*pilA* Δ*fliC* Δ*mvaT* Δ*cupA6* Δ*cupE1-2* | Deletion of *pilA* (PA4525); *fliC* (PA1092); *mvaT* (PA4315); *cupA6* (PA2133) and *cupE1-2* (PA4648-9) | This study |
| PAO1 Δ*pilA* Δ*fliC* Δ*mvaT* Δ*cupA6* Δc*upE1-2 cupE*-complemented | Complementation of *cupE1-2* | This study |
| PAO1 Δ*pilA* Δ*fliC* Δ*mvaT* Δ*cupA6 cupE*1 AGATSST | Site directed mutation of TTTTSST to AGATSST in CupE1 | This study |

**References**

1. Herrero M, de Lorenzo V, Timmis KN. Transposon vectors containing non-antibiotic resistance selection markers for cloning and stable chromosomal insertion of foreign genes in gram-negative bacteria. Journal of Bacteriology. 1990;172(11):6557-67.
