# Supplementary material for: Architecture of the biofilm-associated archaic Chaperone-Usher pilus CupE from Pseudomonas aeruginosa
Source: PLoS Pathog. 2023 Apr 14;19(4):e1011177. doi: 10.1371/journal.ppat.1011177 (PMC10104325; doi:10.1371/journal.ppat.1011177)
Supplement: S5 Table — For the complete assignment of NCBI gene loci to CUP gene clusters for every strain of P. aeruginosa of the Pseudomonas Genome Database, please refer to supplementary data file (S1 Data) additionally included with the manuscript. (DOCX) [file ppat.1011177.s013.docx]

| **Strain** | **Pilin subunits** | | **Chaperone** | **Usher** | **Adhesin** | **Phosphodiesterase** |
| --- | --- | --- | --- | --- | --- | --- |
| **CupA-like** | | | | | | |
| *P. aeruginosa*  PAO1 | | WP_010895601.1 | WP_003088863.1,  WP_003113635.1 | WP_003102159.1 | WP_003113634.1 | WP_003113636.1 |
| *P. aeruginosa*  PAK | | WP_003120281.1 | WP_003088863.1,  WP_003113635.1 | WP_016253472.1 | WP_003102161.1 | WP_003109869.1 |
| *P. aeruginosa*  UCBPP-PA14 | | WP_003120281.1 | WP_003088863.1,  WP_003113635.1 | WP_003139580.1 | WP_003139578.1 | WP_003109869.1 |
| *P. aeruginosa*  PA7 | | - | - | - | - | - |
| *P. fluorescens*  ATCC_13525 | | WP_053255556.1 | WP_053255557.1 | WP_053255761.1 | WP_053255558.1 | WP_053255559.1 |
| *P. putida*  NBRC_14164 | | - | - | - | - | - |
| *P. stutzeri*  PM101005 | | WP_158186206.1,  WP_158186207.1,  WP_158186208.1 | WP_158186209.1,  WP_158186212.1 | WP_158186210.1 | WP_158186211.1 | - |
| *Y. pestis*  CO92 | | WP_002210846.1 | WP_002215118.1,  WP_002210843.1 | WP_002210844.1 | WP_002215120.1 |  |
| *Y. pestis*  CO92 | | WP_002211997.1 | WP_002211998.1,  WP_002212001.1 | WP_002211999.1 | WP_002355452.1 |  |
| *A. baumannii*  *i*ATCC 19606 | | WP_000713424.1 | WP_000898886.1 | WP_005144067.1 | WP_000738483.1 |  |
| *B. pseudomallei* K96243 | | WP_004521426.1 | WP_004527078.1 | WP_009937314.1 | WP_004534800.1 |  |
| *B. pseudomallei* K96243 | | WP_011205334.1,  WP_004529637.1 | WP_004523705.1 | WP_162835921.1 | - |  |
| **CupE-like** | | | | | | |
| *P. aeruginosa*  PAO1 | | WP_003099340.1,  WP_003146046.1,  WP_003099337.1 | WP_003114703.1 | WP_003114701.1 | WP_003099330.1 |  |
| *P. aeruginosa*  PAK | | WP_003117437.1,  WP_003135089.1,  WP_004352708.1 | WP_010793771.1 | WP_010793770.1 | WP_003117435.1 |  |
| *P. aeruginosa*  UCBPP-PA14 | | WP_003099340.1,  WP_003135089.1,  WP_003141616.1 | WP_003141617.1 | WP_003141618.1 | WP_003099330.1 |  |
| *P. aeruginosa*  PA7 | | WP_003149934.1,  WP_023442800.1,  WP_049792051.1 | WP_003149928.1 | WP_012077377.1 | WP_012077378.1 |  |
| *P. fluorescens*  ATCC_13525 | | WP_172900440.1,  WP_053256937.1,  WP_053257999.1,  WP_053256936.1 | WP_053256935.1 | WP_053256934.1 | WP_053257998.1 |  |
| *P. putida*  NBRC_14164 | | WP_041167793.1,  WP_016500513.1,  WP_016500512.1,  WP_016500511.1 | WP_016500510.1 | WP_016500509.1 | WP_016500508.1 |  |
| *P. stutzeri*  PM101005 | | - | - | - | - |  |
| *Y. pestis*  CO92 | | WP_002210852.1,  WP_002216613.1,  WP_002216611.1 | WP_002210856.1 | WP_002210857.1 | WP_002210858.1 |  |
| *A. baumannii*  ATCC 19606 | | WP_000790106.1,  WP_000577018.1,  WP_000876487.1 | WP_001983622.1 | WP_000603317.1 | WP_001022741.1 |  |
| *B. pseudomallei* K96243 | | WP_004526781.1,  WP_004526782.1,  WP_004526783.1 | WP_004526784.1 | WP_045606346.1 | WP_004196717.1 |  |
| *B. pseudomallei* K96243 | | WP_004526328.1 | WP_004191842.1 | WP_230297660.1 | WP_004556779.1 |  |
