# Supplementary material for: Architecture of the biofilm-associated archaic Chaperone-Usher pilus CupE from Pseudomonas aeruginosa
Source: PLoS Pathog. 2023 Apr 14;19(4):e1011177. doi: 10.1371/journal.ppat.1011177 (PMC10104325; doi:10.1371/journal.ppat.1011177)
Supplement: Attachment — Submitted filename: 126396_1_rebuttal_2385078_rdglf8.pdf [file ppat.1011177.s017.pdf]

Revision Plan

Review COMMONS

Manuscript number: RC-2022-01430
Corresponding author(s): Tanmay, Bharat

[The "revision plan" should delineate the revisions that authors intend to carry out in response to the points raised by the referees. It also provides the authors with the opportunity to explain their view of the paper and of the referee reports.

The document is important for the editors of affiliate journals when they make a first decision on the transferred manuscript. It will also be useful to readers of the reprint and help them to obtain a balanced view of the paper.

If you wish to submit a full revision, please use our "Full Revision" template. ***It is important to use the appropriate template to clearly inform the editors of your intentions.****]*

1. General Statements [optional]

This section is optional. Insert here any general statements you wish to make about the goal of the study or about the reviews.

We thank the Review Commons editor for handling of the manuscript and the reviewers for their time and expertise, which have been greatly beneficial to the manuscript.

We are pleased to read that all reviewers rate the structural work highly, which is at the core of this study. Providing first insights into the molecular architecture of an archaic CUP pilus is the main goal of this manuscript. We provide a point-by-point response to additional reviewer comments in the respective categories below.

2. Description of the planned revisions

Insert here a point-by-point reply that explains what revisions, additional experimentations and analyses are planned to address the points raised by the referees.

Reviewer #1 (authors' response in blue):

2. In Ln 171-3, the authors write: "Since GAG is a typical flexible linker motif (Robinson and Sauer, 1998), we hypothesized that flexibility around this hinge might be a key feature of the CupE pilus. Consistent with this hypothesis, curvature within the pilus was observed in a subset of two-dimensional class averages (Figure S3B)."
There is no data supporting the role of GAG in creating a hinge region or creating flexibility in the CupE fibers. The authors use CupE pilus curvature observed in the cryoET maps as the main argument to document CupE flexibility. This does not really allow a quantitative assessment of fiber stiffness and flexibility. In vitro and in situ CupE fibers appear to retain their zig-zag architecture, implying a significant level of axial stiffness. Can the authors provide a more systematic comparison or quantitative assessment of CupE pilus flexibility? This should be compared with polyadhesins such as Afa/Dr, Saf, Caf1, F4 pili (i.e. gamma clade classical CUPs).

We thank the reviewer for this comment. We believe that the various curvatures adopted by CupE pili on cells must imply that subunit arrangement within filament is somewhat flexible, and we strongly suspect the GAG motif at the interface is important for this flexibility. However, we agree that our data does not directly show that the GAG motif acts as a hinge. We will thus soften this message within the manuscript text.

Regarding a systematic comparison: As requested by the reviewer, we will provide a more quantitative assessment of the ranges of curvatures adopted by CupE pili in tomograms. As suggested, we will compare CupE to linear gamma-clade CUP proteins, particularly Saf, for which quantitative information has been reported in negative stain electron microscopy (Salih *et al*, 2008). We will also perform sequence analysis to check for the presence of a comparable GAG linker motif in the gamma-clade CUP proteins.

3. On the subject of axial stiffness in CupE pili, the loop containing residue 62-69 appears to form a major contributor to the zig-zag intersubunit contacts. It would be interesting to observe mutants in this loop and document the effect on CupE flexibility as well as CupE function. Do the authors have a functional readout for CupE activity, like biofilm formation, cell clustering or adherence?

Indeed, the mentioned residues appear to form a significant part of the inter-subunit interface. Our first plan will be to perform detailed bioinformatic analyses to check for conservation of these residues in other described CUP systems with higher rigidity. Doing a mutational analysis of such important core residues might be problematic due to complete loss of filament assembly in

mutants, but we are happy to perform mutational analysis if the bioinformatics provides some clear clues.

4. In the paragraph spanning Lns 174 - 188 the authors investigate the nature of an unidentified density at the height of the TTTTSST loop. Mutagenesis convincingly shows the presence of a posttranslational modification in this region. Was MS analysis tried to try identify the nature of this PTM?

We agree that this is an interesting question. As requested, we will perform additional mass spectrometry experiments to try and identify the nature of this post-translational modification.

Reviewer #2:

Lines 139-140: the authors state that the presence of CupE1 in the pili was confirmed by peptide fingerprint MS. Did the MS results also confirm the presence of other pilus subunits CupE2, CupE3 and CupE6?

Good point. We will perform additional mass spectrometric analyses to probe the presence of these proteins in the revised manuscript. Thus far, our mass spectrometry experiments have been limited to the target sequence of CupE1.

introduction and discussion: is the order of the subunits in the cupE pilus known? The bioinformatic analyses suggest that the adhesion CupE6 can undergo donor strand exchange with CupE1. It is also suggested that subunits CupE2 and CupE3 can form homopolymers. It is unclear whether the authors suggest that CupE2 and CupE3 form pili on their own or whether they suggest that these subunits are incorporated into the pilus together with CupE1 and the adhesin CupE6. Is it known where CupE2 and CupE3 subunits are incorporated into the pilus and in which order? Since their Alpha fold predictions suggest that CupE1 can undergo DSE with CupE6, did the authors also try to form model complexes between CupE1 and CupE2 as well as CupE3 using alpha fold? Would it be possible to derive a subunit order using alpha fold modelling?

During the secretion of CUP systems, the adhesin subunit (here CupE6) is generally secreted first. No structural or functional information on CupE2 and CupE3 has been reported yet. Previous studies have suggested that CupE2 and CupE3 have significant homology to CupE1, and we show here, through structural prediction, that they could also undergo donor strand exchange, suggesting that they might form part of the CupE filaments. Interestingly, previous work shows that deletion of CupE2, but not of CupE3, greatly reduces biofilm formation (Giraud *et al*, 2011).

Unfortunately, we do not think that we can clearly derive a subunit order for CupE1/CupE2/CupE3 using Alphafold modeling. Nevertheless, we indeed performed AF2 prediction for complexes

between CupE1 and CupE2, and CupE1 and CupE3. When predicting a multimer of two CupE1 and two CupE2 subunits, this results in a CupE1-CupE2-CupE1-CupE2 arrangement within the predicted model. We will add these models to the revised version of the manuscript, which will add to the discussion of the revised manuscript.

did the authors test whether the putative post-translation modification affects biofilm formation or stability. Does the GAG pilus variant also form mesh like structures like the Wt pilus?

In the revised manuscript, we will perform a crystal violet assay of biofilm formation that includes the mutant strain compared with the base strain to test the effect of the mutation of biofilm formation to answer the referee's query.

Reviewer #3:

L154 Did the authors carry out a systematic comparison of the CupE1 structure with other CUP pilins (eg by overlay of structures and calculations of rmsd values?) They look to be very similar but it would be useful to try to quantify this.

We agree that this would be a useful analysis and in the revised manuscript, we will provide structural comparisons of our CupE1 structure with other CUP pilin subunits, including the classical rod-like PapA (Hospenthal *et al*, 2016) and monomeric CsuA/B (Pakharukova *et al*, 2018).

L172 The curvature not very precisely described, given its importance to the manuscript. Can an angle be estimated?

As per request, we will provide a quantitative description of curvature values in the revised manuscript.

L183 Did the mass spec analysis of CupE1 preparation indicate any evidence for glycosylation, as suggested in line 183

We will perform additional mass spectrometry experiments aimed at identifying the glycosylation (also requested by reviewer 1 above).

3. Description of the revisions that have already been incorporated in the transferred manuscript

Please insert a point-by-point reply describing the revisions that were already carried out and included in the transferred manuscript. If no revisions have been carried out yet, please leave this section empty.

Reviewer #1:

1. Ln 132-3. Deletion of CupA6 is said to increase the expression of thin surface filaments later identified as CupE. Fig. S1 documents the presence of thin filaments, but no data are shown to allow a quantitative claim on Cup expression. The upregulation of CupE could be document by qPCR and/or Western blotting of whole cells or whole cell lysates. Based on current data, the effect of CupA6 remains anecdotal.

The original manuscript included a quantification of pili in 30 micrographs of Δ*cupA6* versus a control strain without the mutation; we detected 188 fibers in 30 micrographs Δ*cupA6* strain versus 60 fibers in 30 micrographs of the control. We realize that this may be easily overlooked as it is only mentioned in the figure caption. We now include this in the main text, and have adjusted the wording to better reflect this:

> "Indeed, deletion of the *cupA6* phosphodiesterase resulted in increased expression of thin cell surface filaments, as quantified via negative stain electron microscopy, with 188 fibres detected on cells of a Δ*cupA6* strain versus 60 fibres detected in a control over 30 randomly acquired micrographs (Figure S1A, Methods)."

5. In the abstract and in several places throughout the text, the authors draw quite strong functional conclusions on the specifics of the CupE pilus architecture. For example Ln 311-3 "Our study shows that the flexibility of such pili may be a key feature helping them adapt to the complex three-dimensional biofilm environment, allowing them to promote biofilm formation efficiently". There is no data to support such claims and the authors are encouraged to seek an experimental readout of CupE function and use mutant CupE to address the implications of key elements maintaining CupE architecture and axial, rotational or bending stiffness.

We agree that our structural work is not sufficient to prove this point and therefore we have toned down this message within the manuscript.

However, our study provides a first structural basis for future studies on archaic CUP pili. See also our response to comment 3 regarding the characterization of 'key elements maintaining CupE architecture and axial, rotational or bending stiffness' through mutation.

Reviewer #2:

a sentence is missing in the introduction that clarifies whether the subunit composition of the cupE pilus is known or not. Lines 95-97 state that the cup operon is formed by CupE1-CupE6. This indicates that CupE2 and CupE3 are part of the pilus. However, in the abstract the authors state that the "CupE pili consist of CupE1 subunits".

As requested, we have adjusted the abstract to clarify this. We now state -

> "We show that CupE1 subunits within the pilus are arranged in a zigzag architecture"

Lines 77-79: the authors state that structural information on non-classical CUP systems is scarce. It would be nice to mention what is known.

We now elaborate on this:

> "However, available structural information on non-classical CUP pili is scarce, being limited to a crystal structures of a pilin of the archaic Csu pilus (Pakharukova *et al*, 2015)."

figure numbering is not consistent between lines 161-164: after Fig S3A follows Fig S4

We have now corrected this. Sorry about this.

Reviewer #3:

L151 'large mesh-like bodies'- Fig S1D; is this an artefact of concentration of purified pili or might it have some physiological significance?

This is an interesting question – we only wanted to report the propensity of CupE pili to form such structures. It seems plausible that bundling of pili may have a function in the intact biofilm, where significant molecular crowding in the extracellular polymeric matrix may induce such bundling. As mentioned in the discussion section in the original manuscript, determining this will be the goal of future studies:

> "While we study CupE pili on cells, an intrinsic limitation of our system is that single cells expressing CupE do not fully recapture the molecular sociology and crowding conditions within intact biofilms. Interaction partners of the pilus are hence unknown and warrant the focus of future imaging efforts. Since we have observed isolated CupE pili forming regular mesh-like arrays in cryo-EM images (Figure S1D), this might indicate lateral interactions of pili may occur in the crowded conditions of the biofilm matrix, similar to other biofilm matrix fiber systems (Boehning *et al*, 2022; Tarafder *et al*, 2020). Further studies on native cellular

systems – i.e., biofilms - will be required to determine the exact mode of interaction of CupE pili with other extracellular matrix components and cells.”

4. Description of analyses that authors prefer not to carry out

Please include a point-by-point response explaining why some of the requested data or additional analyses might not be necessary or cannot be provided within the scope of a revision. This can be due to time or resource limitations or in case of disagreement about the necessity of such additional data given the scope of the study. Please leave empty if not applicable.

We are happy to carry out all the inquiries requested by the reviewers as detailed above with one small caveat - we are slightly unsure about a point raised by reviewer 1 about mutations in the inter-subunit interface. In our experience of working with filamentous proteins, these mutations will usually disrupt filament formation, not leading to any discernable information about function. We will only pursue these mutations if the bioinformatic analyses provide us with a clear clue on what to mutate, based on a comparison with other CUP pili.

References

Boehning J, Ghrayeb M, Pedebos C, Abbas D, Khalid S, Chai L, Bharat T (2022) Molecular architecture of the TasA biofilm scaffold in *Bacillus subtilis*. *bioRxiv*
Giraud C, Bernard CS, Calderon V, Yang L, Filloux A, Molin S, Fichant G, Bordi C, de Bentzmann S (2011) The PprA–PprB two-component system activates CupE, the first non-archetypal *Pseudomonas aeruginosa* chaperone–usher pathway system assembling fimbriae. *Environmental Microbiology* 13: 666-683
Hospenthal MK, Redzej A, Dodson K, Ukleja M, Frenz B, Rodrigues C, Hultgren SJ, DiMaio F, Egelman EH, Waksman G (2016) Structure of a chaperone-usher pilus reveals the molecular basis of rod uncoiling. *Cell* 164: 269-278
Pakharukova N, Garnett JA, Tuittila M, Paavilainen S, Diallo M, Xu Y, Matthews SJ, Zavialov AV (2015) Structural insight into archaic and alternative chaperone-usher pathways reveals a novel mechanism of pilus biogenesis. *PLoS Pathogens* 11: e1005269
Pakharukova N, Tuittila M, Paavilainen S, Malmi H, Parilova O, Teneberg S, Knight SD, Zavialov AV (2018) Structural basis for *Acinetobacter baumannii* biofilm formation. *Proceedings of the National Academy of Sciences* 115: 5558-5563
Salih O, Remaut H, Waksman G, Orlova EV (2008) Structural analysis of the Saf pilus by electron microscopy and image processing. *Journal of molecular biology* 379: 174-187
Tarafder AK, von Kügelgen A, Mellul AJ, Schulze U, Aarts DG, Bharat TA (2020) Phage liquid crystalline droplets form occlusive sheaths that encapsulate and protect infectious rod-shaped bacteria. *Proceedings of the National Academy of Sciences* 117: 4724-4731
