# Supplementary material for: Architecture of the biofilm-associated archaic Chaperone-Usher pilus CupE from Pseudomonas aeruginosa
Source: PLoS Pathog. 2023 Apr 14;19(4):e1011177. doi: 10.1371/journal.ppat.1011177 (PMC10104325; doi:10.1371/journal.ppat.1011177)
Supplement: Attachment — Submitted filename: Boehning_etal_revised_Response.pdf [file ppat.1011177.s018.pdf]

<u>Response to reviewers</u>
PPATHOGENS-D-22-01186

Black: Reviewer comments
Orange: Original revision plan
Blue: Implemented revision
Green: Cited manuscript changes

Note: It appears that Reviewer #3 from ReviewCommons corresponds to Reviewer #1 in the PLOS Pathogens review, and vice versa. We have adjusted for this - the Reviewer numbers mentioned here refer to those in the PLOS Pathogens decision email.

Comments by Reviewer #1:

Original reviews (ReviewCommons):
L154 Did the authors carry out a systematic comparison of the CupE1 structure with other CUP pilins (eg by overlay of structures and calculations of rmsd values?) They look to be very similar but it would be useful to try to quantify this.

Original revision plan:
We agree that this would be a useful analysis and in the revised manuscript, we will provide structural comparisons of our CupE1 structure with other CUP pilin subunits, including the classical rod-like PapA (Hospenthal et al., 2016) and monomeric CsuA/B (Pakharukova et al., 2018).

Implemented revision:
We now include a new supplementary figure (Figure S5) with structural comparisons with the Csu pilin from the intact Csu pilus (recently released in Pakharukova et al. (2022)) and *E. coli* PapA (Hospenthal et al., 2016) in the revised manuscript. Interestingly, RMSD between CsuA/B and CupE is relatively low, 1.55 Å, compared to the RMSD between PapA and CupE, which is 5.5 Å.

Original reviews (ReviewCommons) and response to revision plan (PLOS Pathogens):
L172 The curvature not very precisely described, given its importance to the manuscript. Can an angle be estimated?

Original revision plan:
As per request, we will provide a quantitative description of curvature values in the revised manuscript.

Implemented revision:
We now quantify the angles between CupE subunits within a tomogram to show the curvature between subunits within the pilus (Figure S6E). To do this, we have segmented the pilus within the tomogram, and on the path of the pilus calculated deviation from the helical axis every 33.1 Å, corresponding to the CupE helical rise. Our analysis shows that angles can deviate up to 15° from helical axis. This indicates local flexibility of the CupE pilus compared to rod-like CUP pili that are stiff. Nevertheless, due to the reviewer’s concerns we reworded cautiously the mentioning of flexibility within the revised manuscript.

Original reviews (ReviewCommons) and response to revision plan (PLOS Pathogens):
L183 Did the mass spec analysis of CupE1 preparation indicate any evidence for glycosylation, as suggested in line 183

Original revision plan:
We will perform additional mass spectrometry experiments aimed at identifying the glycosylation (also requested by reviewer 1 above).

Implemented revision:
We have now performed intact MS analysis, which predominantly yielded a species that corresponds to the size of an unmodified CupE1 subunit. While we can detect another species at 206 Da larger, which might correspond a sugar moiety, we cannot conclusively assign its chemical identity despite repeated attempts. We have accordingly toned down our messaging in the manuscript, as the extra density might well be some other unknown bound molecules.

Cited manuscript changes:
L206: "While disassembly into monomers followed by intact mass spectrometric analysis allowed us to detect a molecular species 206 Da larger than the expected mass of the CupE1 monomer (Figure S3G), predominantly unmodified CupE1 protein was observed. Thus, the chemical identity of the extra density near the threonine-rich loop cannot be ascertained at this stage."

Original reviews (ReviewCommons) and response to revision plan (PLOS Pathogens):
L155 'large mesh-like bodies'- Fig S1D; is this an artefact of concentration of purified pili or might it have some physiological significance?

Implemented revision:
Unfortunately, this is hard to confirm either way; we could imagine both are possible, but cryo-ET imaging of native biofilms will be required to further study this. This is mentioned in the Discussion:

Cited manuscript changes:
L347: "The interaction partners of the pilus are hence unknown and warrant the focus of future imaging efforts. Since we observed isolated CupE pili forming regular mesh-like arrays in cryo-EM images (Figure S1D), this might indicate that lateral interactions of pili may occur in the crowded conditions of the biofilm matrix, similar to other biofilm matrix fiber systems (Tarafder et al., 2020, Böhning et al., 2022b, Böhning et al., 2022a)."

Comments by Reviewer #2

Original reviews (ReviewCommons):

Lines 139-140: the authors state that the presence of CupE1 in the pili was confirmed by peptide fingerprint MS. Did the MS results also confirm the presence of other pilus subunits CupE2, CupE3 and CupE6?

Original revision plan:

Good point. We will perform additional mass spectrometric analyses to probe the presence of these proteins in the revised manuscript. Thus far, our mass spectrometry experiments have been limited to the target sequence of CupE1.

Implemented revision:

We have performed peptide fingerprinting MS of our sample multiple times now, which has sequence coverage of ~50% of mature CupE1, but we have not been able to detect any subunits other than CupE1. We can only suspect this might be a sensitivity issue – for >3000 subunits of CupE1, one would expect only one of the CupE6 tip subunits.

Original reviews (ReviewCommons):

introduction and discussion: is the order of the subunits in the cupE pilus known? The bioinformatic analyses suggest that the adhesion CupE6 can undergo donor strand exchange with CupE1. It is also suggested that subunits CupE2 and CupE3 can form homopolymers. It is unclear whether the authors suggest that CupE2 and CupE3 form pili on their own or whether they suggest that these subunits are incorporated into the pilus together with CupE1 and the adhesin CupE6. Is it known where CupE2 and CupE3 subunits are incorporated into the pilus and in which order? Since their Alpha fold predictions suggest that CupE1 can undergo DSE with CupE6, did the authors also try to form model complexes between CupE1 and CupE2 as well as CupE3 using alpha fold? Would it be possible to derive a subunit order using alpha fold modelling?

Original revision plan:

Sadly, we do not think that we can clearly derive a subunit order for CupE1/CupE2/CupE3 using Alphafold modeling. Nevertheless, we indeed performed AF2 prediction for complexes between CupE1 and CupE2, and CupE1 and CupE3. When predicting a multimer of two CupE1 and two CupE2 subunits, this results in a CupE1-CupE2-CupE1-CupE2 arrangement within the predicted model. We will add these models to the revised version of the manuscript, which will add to the discussion of the revised manuscript.

Implemented revision:

We have now performed AlphaFold modelling of filaments made of two CupE1 and CupE2 subunits, and two CupE1 and two CupE3 subunits, respectively (new panels in Figure S7-8). We noticed that, depending on the settings used for modelling, arrangements of E1-E2-E1-E2 and E1-E1-E2-E2 could be obtained. Using the same settings as for the initial models, we obtain E2-E2-E1-E1 and E3-E3-E1-E1 arrangements (Figure S7). Beyond the fact that these filament arrangements appear possible, we cannot say more. We further discuss the possibilities of subunit arrangement in the revised manuscript:

Cited manuscript changes:

L325: “AlphaFold structure predictions for minor CupE subunits CupE2 and CupE3 suggest that they can also form filaments through donor-strand exchange. While both homofilaments and heterofilaments with CupE1 are predicted to be possible (Figure S7), only CupE1 homofilaments appear to form the more compact zigzag architecture. However, it is unclear whether the minor pilins CupE2 and CupE3 form homofilaments at certain sections of the pilus, similar to the tips in the classical Type 1 and P pili (Waksman and Hultgren, 2009), or whether they are sub-stoichiometrically embedded between CupE1 subunits. Other possibilities are that they functionalize pili or fulfil an undetermined function in pilus assembly.”

Response to revision plan (PLOS Pathogens):
As mentioned in my previous comments, the manuscript needs a couple of sentences that clarify what is known and what is not known about subunit composition and subunit order of CupE pili.

Implemented revision:
We now added additional text to the introduction. Unfortunately, very little is known about the subunits:

Cited manuscript changes:
L100: “The *cupE* gene cluster encodes one major pilin subunit (CupE1) that is the main component of the pilus, two minor pilin subunits (CupE2, CupE3) whose arrangement or function within the pilus is unknown, a chaperone (CupE4), an usher (CupE5), and a tip adhesin protein (CupE6).”

Original reviews (ReviewCommons):
did the authors test whether the putative post-translation modification affects biofilm formation or stability. Does the GAG pilus variant also form mesh like structures like the Wt pilus?

Original revision plan:
In the revised manuscript, we will perform a crystal violet assay of biofilm formation that includes the mutant strain compared with the base strain to test the effect of the mutation of biofilm formation to answer the referee’s query.

Implemented revision:
We have now performed a biofilm assay of the loop mutant strain versus the background strain according to O'Toole (2011) with six replicates per condition using M63 medium with casamino acids (O'Toole, 2011); we detect no decrease in biofilm formation propensity in the mutant (Figure R1). However, the strains we employ in the study have a Δ*fliC* Δ*pilA* background, which is helpful for purification as it prevents contamination by pili and flagella, but are defective in biofilm development compared to WT, consistent with previous literature (Klausen et al., 2003). Given that we were thus far not able to detect modification using mass spectrometry, we have toned down messaging around the post-translational modification.

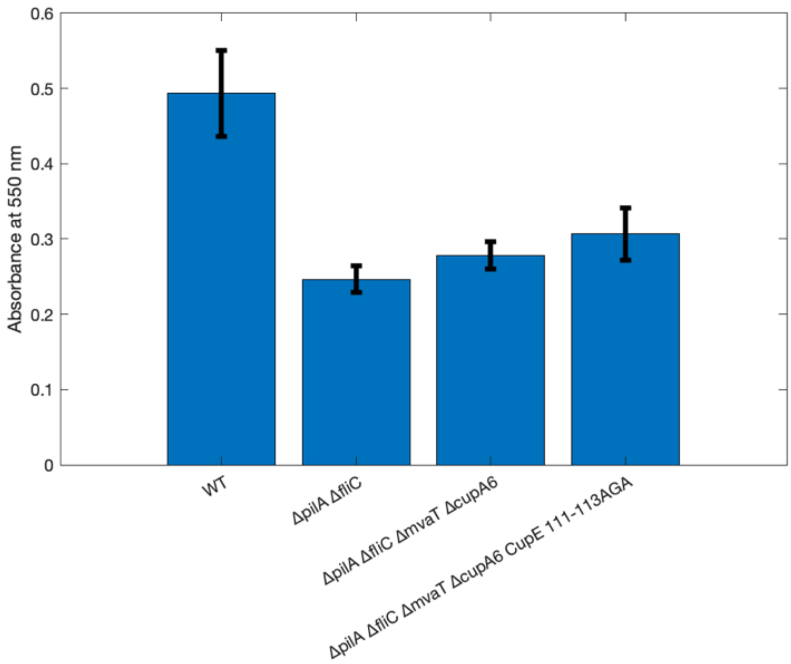

Figure R1: Biofilm assay of *P. aeruginosa* strains according to showing biofilm formation as a measure of crystal violet absorbance at 550 nm. Six biological replicates were used per condition; error bars show standard deviation. Cells were grown in M63 minimal media supplemented with casamino acids as the carbon source (O'Toole, 2011). All strains with a Δ*pilA* Δ*fliC* showed reduced biofilm formation compared to WT.

Comments by Reviewer #3

Original reviews (ReviewCommons):
2. In Ln 171-3, the authors write: "Since GAG is a typical flexible linker motif (Robinson and Sauer, 1998), we hypothesized that flexibility around this hinge might be a key feature of the CupE pilus. Consistent with this hypothesis, curvature within the pilus was observed in a subset of two-dimensional class averages (Figure S3B)."
There is no data supporting the role of GAG in creating a hinge region or creating flexibility in the CupE fibers. The authors use CupE pilus curvature observed in the cryoET maps as the main argument to document CupE flexibility. This does not really allow a quantitative assessment of fiber stiffness and flexibility. In vitro and in situ CupE fibers appear to retain their zig-zag architecture, implying a significant level of axial stiffness. Can the authors provide a more systematic comparison or quantitative assessment of CupE pilus flexibility? This should be compared with polyadhesins such as Afa/Dr, Saf, Caf1, F4 pili (i.e. gamma clade classical CUPs).

Original revision plan:
We thank the reviewer for this comment. We believe that the various curvatures adopted by CupE pili on cells must imply that subunit arrangement within filament is somewhat flexible, and we strongly suspect the GAG motif at the interface is important for this flexibility. However, we agree that our data does not directly show that the GAG motif acts as a hinge. We will thus soften this message within the manuscript text.

Regarding a systematic comparison: As requested by the reviewer, we will provide a more quantitative assessment of the ranges of curvatures adopted by CupE pili in tomograms. As suggested, we will compare CupE to linear gamma-clade CUP proteins, particularly Saf, for which quantitative information has been reported in negative stain electron microscopy (Salih et al., 2008). We will also perform sequence analysis to check for the presence of a comparable GAG linker motif in the gamma-clade CUP proteins.

Reviewer response to revision plan and initial revisions (PLOS Pathogens):
In their new manuscript, the author respond to comments and suggestions I made in the context of an earlier submission. I am supportive of publication of the study, but remain of the opinion that the extend of flexibility in the CupE1 polymer is excessively emphasized. This is done in abstract (Ln 31-35), main text (Ln 216-218) and discussion (Ln 293-297; 316-319), and is suggested to be a functionally ‘key feature’ and to ‘contrast’ with the archaic Csu pilus of A. baumannii, which was recently described to assemble into a supereleastic zig-zag spring (Pakharukova et al. 2022). The reader is left with the image that CupE pili would be “akin a rope” that is flexible and wraps around other objects. I see no evidence for this strong emphasis. A couple of arguments to substantiate my opinion:

1. The cryoET data do show the presence of long range curvature in the CupE pili, but there is no real quantitative measure of the flexibility of the CupE1 polymer. Based on the images provided, the persistence length still looks quite large and the subtomogram averaging shows the fibers maintain the zig-zag architecture. Whilst this does show at least some flexibility in the subunit-subunit contact, there is no data to say this involves the GAG linker and the way now formulated may give the reader the impression that the contact between CupE1 subunits is essentially flexible, which is clearly not the case.

Implemented revision:
We now quantify the angles between CupE subunits within a tomogram to give a measure of curvature adopted by subunits within the pilus (new panel, Figure S6E). This analysis suggests that subunit-subunit angles can deviate up to 15° from the helical axis of our cryo-EM structure. While this indicates long-range flexibility of the CupE pilus compared to rod-like pili that are rather stiff (Hospenthal et al., 2016), we agree with the reviewer that the CupE pilus is clearly not as flexible as Saf and Caf1, which are considerably more flexible *in vitro* and show even higher angles between subunits (up to 38° angles in the case of Saf1 (Salih et al., 2008)). We have commented on this in the manuscript now.

Cited manuscript changes:
L229: "We estimated the local curvature of CupE pili in cellular tomograms and discovered local deviation from the helical axis of up to 15º per subunit (Figure S6E). This observed ability of CupE pili to locally adopt higher curvature contrasts with classical, tubular CUP pili, which are comparatively rigid assemblies (Hospenthal et al., 2016). At the same time, however, it also differs from γ-clade linear Chaperone-Usher fibres, such as the Caf1 and Saf pilus that were found to have extremely low persistence lengths and even higher angles between subunits (Salih et al., 2008, Soliakov et al., 2010)."

The CupE pilus, as mentioned by the reviewer, only locally adopts increased curvature. We differentiate this more clearly in the revised version of the manuscript, toning down mentions of 'flexibility' in several instances. We also remove mentions of a role of the GAG linker in enabling flexibility in the revised manuscript. We further removed the rope analogy the reviewer mentioned and provide further analyses of inter-subunit interfaces in CUP pili (Figure S5).

2. Looking at the presented structure, it appears the "clinch contact" shown to underlie zig-zag architecture and the superelastic nature of the Csu pilus is highly similar, if not conserved, in the CupE1 zig-zag architecture: i.e. Figure 2D the loop S62-V69 in subunit n and the pocket formed by G40 – W55 in subunit n+1.
A more detailed comparison between both structures should be included in the supplementary Figures and discussion.
Again, the subtomographic averaging shows this clinch contact remains essentially intact, but does allow long range flexibility in the fibers. It seems quite plausible to me that the spring-like and superelastic nature of Csu will proof to be conserved in CupE1, albeit with a lesser stiffness of the fiber.

Implemented revision:
We agree that the super-elasticity shown for the Csu pilus is likely conserved in the CupE pilus and now mention this in the manuscript. Now that a PDB file for the Csu pilus has been released after the journal publication of the corresponding manuscript from the Zavialov lab (Pakharukova et al., 2022), we provide a new supplementary figure (Figure S5) that compares the pilin structures and interactions between subunits in the Csu and CupE pilus.

Interestingly, while the CupE1 and CsuA/B pilins are highly homologous, it appears that there is a tighter packing of subunits in the Csu pilus, with more interactions between subsequent subunits, resulting in a rise of ~28 Å for Csu versus a rise of ~33 Å for CupE. The resultingly increased subunit contacts may explain why the Csu pilus is stiffer than the CupE

pilus, which has less interactions between subunits, potentially allowing for more flexibility. We address this now in the revised manuscript:

Cited manuscript changes:
L318: “Interestingly, it was found in optical tweezer experiments that the Csu pilus can be extended to almost twice its length along the helical axis by breaking of the subunit interface contacts (Pakharukova et al., 2022). Given that CupE shares a similar architecture, it seems likely that it shares this super-elasticity, suggesting it may be a common feature of archaic CUP pili. We propose that the lateral curvature of the CupE pilus observed in the cellular environment may stem from the same properties, i.e., the result of breaking some or all non-donor-strand subunit interface contacts.”

3. The alphafold2 modelling of homopolymers of the different CupE subunits suggests that the potential clinch contact and resulting zig-zag architecture are unique to CupE1 (a feature conserved in the major subunits of archaic CUPs ?) and that the contacts zones between the minor CupE subunits (E2, E3 and E6) are less ordered. That would bring forward a picture where the zig-zag architecture in the archaic main pilus subunits is conserved, forming the equivalent of the more rigid helical packing in the rod of classical (i.e. gamma and pi clade) pilus systems, and that the minor subunits may form the equivalent of the more flexible tip fibrillae in classical pili, or main form hinge regions if incorporated within the polymer of the major subunit. The role of the minor subunits remains to be confirmed, but the authors could include potential scenarios in their discussion.

Implemented revision:
We agree that these are plausible possibilities and discuss them in the revised manuscript:

Cited manuscript changes:
L325: “AlphaFold structure predictions for minor CupE subunits CupE2 and CupE3 suggest that they can also form filaments through donor-strand exchange. While both homofilaments and heterofilaments with CupE1 are predicted to be possible (Figure S7), only CupE1 homofilaments appear to form the more compact zigzag architecture. However, it is unclear whether the minor pilins CupE2 and CupE3 form homofilaments at certain sections of the pilus, similar to the tips in the classical Type 1 and P pili (Waksman and Hultgren, 2009), or whether they are sub-stoichiometrically embedded between CupE1 subunits. Other possibilities are that they functionalize pili or fulfil an undetermined function in pilus assembly.”

4. There is no data in the manuscript that allow any claim regarding the functional importance of the CupE1 curvature described in the manuscript. I think this should be discussed with more caution than is currently done.

Implemented revision:
We have toned down respective language in the revised manuscript. Please also see our response to another similar point above.

Original reviews (Review Commons):
On the subject of axial stiffness in CupE pili, the loop containing residue 62-69 appears to form a major contributor to the zig-zag intersubunit contacts. It would be interesting to observe mutants in this loop and document the effect on CupE flexibility as well as CupE function. Do

the authors have a functional readout for CupE activity, like biofilm formation, cell clustering or adherence?

Original revision plan:
[..] we are slightly unsure about a point raised by reviewer 1 about mutations in the inter-subunit interface. In our experience of working with filamentous proteins, these mutations will usually disrupt filament formation, not leading to any discernable information about function. We will only pursue these mutations if the bioinformatic analyses provide us with a clear clue on what to mutate, based on a comparison with other CUP pili.

Implemented revision:
We have now checked conservation in the residues at the interface between domains (62-69), and, while residues with side chains that interact with the donor strand within the same fold are conserved (L66 and V70) are conserved, it appears that other residues at the domain interface are not conserved. This is exemplified by the alignment shown in Figure S4, and we have now marked these residues within the alignment. We now also mention this in the figure legend:

Cited manuscript changes:
L628: "Residues 62-69, which are located near the subunit-subunit interface, are marked in grey. L66 and V70, which interact with the donor strand within the same subunit fold, are conserved; residues facing the subunit-subunit interface are not conserved."

Original reviews (ReviewCommons):
In the paragraph spanning Lns 174 - 188 the authors investigate the nature of an unidentified density at the height of the TTTTSST loop. Mutagenesis convincingly shows the presence of a posttranslational modification in this region. Was MS analysis tried to try identify the nature of this PTM?

and

Reponse to revision plan (PLOS Pathogens):
- In Ln180 – 194 the authors observed a residual density in a T/S rich loop that is suggestive of post-translational modification, likely O-glycosylation. Mutation of a TTT stretch to GAG results in loss of the residual density confirming the presence of the PTM. The assignment as a candidate glycosylation site seems plausible. Did the MS peptide fingerprint that was performed show the presence of the PTM?

Implemented revision:
We have now performed intact MS analysis, which predominantly yielded a species that corresponds to the size of an unmodified CupE1 subunit. While we can detect another, 206 Da larger species, which might correspond a sugar moiety, we cannot conclusively assign its chemical identity. We have accordingly toned down our messaging in the manuscript, as the extra density might well be some other unknown bound molecules.

Cited manuscript changes:
L206: "While disassembly into monomers followed by intact mass spectrometric analysis allowed us to detect a molecular species 206 Da larger than the expected mass of the CupE1

monomer (Figure S3G), predominantly unmodified CupE1 protein was observed. Thus, the chemical identity of the extra density near the threonine-rich loop cannot be ascertained at this stage."

Reponse to revision plan (PLOS Pathogens):
Ln 63. The authors reference the Hospenthal 2016 review for the donor strand complementation mechanism of subunit-subunit contacts. The primary reference for this principle is Sauer et al. 2002.

Sorry about that - we have now cited the correct source.

References

BOEHNING, J., GHRAYEB, M., PEDEBOS, C., ABBAS, D., KHALID, S., CHAI, L. & BHARAT, T. 2022. Molecular architecture of the TasA biofilm scaffold in *Bacillus subtilis*. *bioRxiv*.

HOSPENTHAL, M. K., REDZEJ, A., DODSON, K., UKLEJA, M., FRENZ, B., RODRIGUES, C., HULTGREN, S. J., DIMAIO, F., EGELMAN, E. H. & WAKSMAN, G. 2016. Structure of a chaperone-usher pilus reveals the molecular basis of rod uncoiling. *Cell,* 164**,** 269-278.

KLAUSEN, M., HEYDORN, A., RAGAS, P., LAMBERTSEN, L., AAES-JØRGENSEN, A., MOLIN, S. & TOLKER-NIELSEN, T. 2003. Biofilm formation by Pseudomonas aeruginosa wild type, flagella and type IV pili mutants. *Molecular microbiology,* 48**,** 1511-1524.

O'TOOLE, G. A. 2011. Microtiter dish biofilm formation assay. *JoVE (Journal of Visualized Experiments)***,** e2437.

PAKHARUKOVA, N., MALMI, H., TUITTILA, M., DAHLBERG, T., GHOSAL, D., CHANG, Y.-W., MYINT, S. L., PAAVILAINEN, S., KNIGHT, S. D. & LAMMINMÄKI, U. 2022. Archaic chaperone–usher pili self-secrete into superelastic zigzag springs. *Nature,* 609**,** 335-340.

PAKHARUKOVA, N., TUITTILA, M., PAAVILAINEN, S., MALMI, H., PARILOVA, O., TENEBERG, S., KNIGHT, S. D. & ZAVIALOV, A. V. 2018. Structural basis for *Acinetobacter baumannii* biofilm formation. *Proceedings of the National Academy of Sciences,* 115**,** 5558-5563.

SALIH, O., REMAUT, H., WAKSMAN, G. & ORLOVA, E. V. 2008. Structural analysis of the Saf pilus by electron microscopy and image processing. *Journal of molecular biology,* 379**,** 174-187.

SOLIAKOV, A., HARRIS, J. R., WATKINSON, A. & LAKEY, J. H. 2010. The structure of Yersinia pestis Caf1 polymer in free and adjuvant bound states. *Vaccine,* 28**,** 5746-5754.

TARAFDER, A. K., VON KÜGELGEN, A., MELLUL, A. J., SCHULZE, U., AARTS, D. G. & BHARAT, T. A. 2020. Phage liquid crystalline droplets form occlusive sheaths that encapsulate and protect infectious rod-shaped bacteria. *Proceedings of the National Academy of Sciences,* 117**,** 4724-4731.

WAKSMAN, G. & HULTGREN, S. J. 2009. Structural biology of the chaperone–usher pathway of pilus biogenesis. *Nature Reviews Microbiology,* 7**,** 765-774.
